# Timing effects in the association between childhood and adolescent bullying victimisation with late adolescence and emerging adulthood depressive symptoms

**DOI:** 10.1007/s00787-026-03011-9

**Published:** 2026-03-23

**Authors:** Sarah Bakirci, Andrew D.A.C. Smith, Jean-Baptiste Pingault

**Affiliations:** 1 Department of Clinical, Educational and Health Psychology, University College London, 26 Bedford Way, London, WC1H 0AP United Kingdom; 2 Mathematics and Statistics Research Group, University of the West of England, 3E020, School of Computing and Creative Technologies, Bristol, BS16 1Q7 United Kingdom

## Abstract

Bullying victimisation during childhood is a significant risk factor for psychopathology, particularly depression, with some studies suggesting lasting associations into adulthood. However, these associations may vary based on developmental timing, i.e., age at exposure. Life course epidemiology proposes three timing hypotheses: (1) accumulation of risk, where additional exposure events increase impact; (2) recency, where proximal experiences have a greater impact than distal ones; and (3) sensitive periods, where specific timing windows increase vulnerability. These hypotheses have yet to be formally tested to better understand how the timing of bullying victimisation affects depression in emerging adulthood. This study explores the relationship between the timing of bullying victimisation and depression symptoms in late adolescence and emerging adulthood. Using data from the Avon Longitudinal Study of Parents and Children (*N* = 6,782), we applied the Structured Life Course Modelling Approach (SLCMA) to test accumulation of risk, recency, and sensitive period hypotheses. Bullying was measured with the Strengths and Difficulties Questionnaire at ages 4, 7, 8, 9, 11, 13, and 16. Depression symptoms were measured with the Short Mood and Feelings Questionnaire (SMFQ) at ages 16.5, 17.5, 21, 22, and 23. Developmental windows were defined as early childhood (0–5 years), middle childhood (6–10 years), early adolescence (11–14 years), and late adolescence (15–17 years). Model selection by SLCMA consistently favoured the recency hypothesis across outcome timepoints, such that exposure to bullying was associated with an increase in SMFQ at all ages between 4 and 16 years, with a greater increase for exposure to bullying at older ages: a unit increase in bullying victimisation score at age 4 was associated with an increase of 0.188 (95% CI: 0.160–0.260) in SMFQ score at age 16.5, while a unit increase at age 16 was associated with an increase of 0.753 (95% CI: 0.640–0.867). Findings suggest that the association between bullying victimisation and depressive symptoms is primarily driven by recency. Early post-exposure intervention is essential to reduce later mental health consequences.

**Supplementary Information:**

The online version contains supplementary material available at 10.1007/s00787-026-03011-9.

## Introduction

Bullying victimisation is a repeated occurrence of physical, verbal, direct or indirect abuse between individuals of the same age group [1]. It typically involves a power imbalance between the perpetrator and the victim [2]. Bullying has a widespread prevalence with a rate of approximately 13% of 11 year olds in 40 European countries reporting being victims [3]. Furthermore, bullying is the most prevalent form of abuse that children face [4].

Bullying victimisation poses a serious risk for the development of psychiatric problems and is thought to have long-lasting associations with physical and emotional health [5]. Adolescents who are frequently bullied are two to three times more likely to develop an anxiety disorder and two times more likely to develop depression than non-bullied adolescents [6–8]. These associations can persist well into adulthood, with some evidence pointing as far as mid-life [5, 9, 10]. Multiple lines of evidence indicate that such associations between bullying victimisation and subsequent anxiety and depression partly reflect the causal effect of bullying victimisation [11, 12].

Among mental health outcomes, bullying is most strongly associated with internalising symptoms, particularly depression [11]. Meta-analytic evidence, including quasi-experimental designs, demonstrates substantial and robust associations between bullying victimisation and increased risk for depressive symptoms [11, 13]. As such, we focus on depressive symptoms to test for timing effects. Although, it is clear that experiencing bullying during childhood is linked to adverse subsequent mental health, not much is known about whether or how the specific timing of experiencing bullying influences later-life mental health.

### Life course hypotheses of bullying on later depression

Life course epidemiology offers a valuable framework for understanding how the timing of experiences might influence later outcomes, using repeated measures of an exposure. In this framework, three hypotheses are central: (1) the accumulation of risk hypothesis posits that the effect of an adverse event, such as bullying victimisation, on an outcome, such as depression, is additive, with each additional exposure increasing the overall risk; (2) the recency hypothesis suggests that more proximal experiences (i.e., recent bullying events) have a stronger effect on an outcome (i.e., adult depression) than those occurring earlier in life; and (3) the sensitive periods hypothesis proposes that there are specific developmental windows during which an adverse exposure, in this case bullying, may have a particularly profound impact on an individual’s vulnerability to an outcome, such as depression [14].

Some studies have found that chronic and repeated bullying across time puts victims at highest psychopathology risk notably for psychotic experiences in later adolescence, which ties into the accumulation of risk hypothesis [15]. Some longitudinal work has also shown dose–response relationships between repeated victimisation and later internalising symptoms [8, 16, 17]. One study found that participants who were bullied at both ages 8 and 10 had a higher association with internalising symptoms at ages 11 and 14 than those who were only bullied at one timepoint [8]. These studies indicate that the accumulation of bullying may be associated with later internalising symptoms. However, no study to our knowledge has formally examined the accumulation of risk of repeated bullying victimisation across both childhood and adolescence and associations to later depression.

Some studies have suggested that early-life exposure to general victimisation increases the risk of re-victimisation and the risk of psychopathology [18–20]. However, it is unclear whether the early exposure or the repeated victimisation is responsible for the increased risk of psychopathology. Furthermore, unmeasured external factors could explain the increased risk of re-victimisation and development of psychopathology more so than the first early incidence of bullying. Albeit not specific to bullying, many studies have identified sensitive periods for childhood adversity in general. A meta-analysis by Schaefer, Cheng, and Dunn [21] including 118 cross-sectional or longitudinal studies determined that, despite 75% of the studies identifying timing effects of child maltreatment, the meta-analysis failed to detect consistent sensitive periods.

There is some evidence for the recency hypothesis and bullying victimisation. A study by Singham [22] found evidence of a fade out of the effects of bullying after the exposure. The study used a twin difference design to examine the impact of bullying victimisation on mental health outcomes concurrently, two years and five years following the exposure. The results demonstrated an immediate impact of bullying on various psychopathology symptoms, notably depression. The impact decreased after two years, fading out completely by the five-year follow-up. These results suggest, not only a causal impact of bullying on mental health, but also a dissipation of the impact of bullying on most mental health outcomes across time, indicating a potential recency life course trend. Overall, although some studies have pointed in the direction of timing effects, none have systematically tested the role timing of bullying victimisation and subsequent depression symptoms.

This study aims to systematically test pre-specified timing hypotheses of accumulation of risk, sensitive periods, and recency of bullying victimisation exposure during childhood and adolescence and depression symptoms in late adolescence following up into emerging adulthood using a longitudinal birth cohort. Given the above evidence, we hypothesise that our results will demonstrate the recency hypothesis is most appropriate for bullying victimisation and the development of depression in emerging adulthood.

### Methods

#### Sample

The Avon Longitudinal Study of Parents and Children (ALSPAC) is a large birth cohort study based in South West England which includes data on mothers, fathers, and children [23, 24]. Pregnant women resident in Avon, UK with expected dates of delivery between 1st April 1991 and 31st December 1992 were invited to take part in the study [23–25]. Of 20,248 eligible, 14,541 were initially enrolled, resulting in 13,988 children alive at 1 year of age. When the cohort children were approximately 7 years old, additional eligible cases who had not joined originally were recruited, increasing the total to 15,447 pregnancies, and 15,658 foetuses. Among these, 14,901 children were alive at 1 year of age. This expanded sample forms the basis for analyses involving data collected after age seven.

Study data were collected and managed using REDCap electronic data capture tools hosted at the University of Bristol [26]. REDCap (Research Electronic Data Capture) is a secure, web-based software platform designed to support data capture for research studies. Please note that the study website contains details of all the data that are available through a fully searchable data dictionary and variable search tool: http://www.bristol.ac.uk/alspac/researchers/our-data/. Ethical approval for the study was obtained from the ALSPAC Ethics and Law Committee and the Local Research Ethics Committees.

Participants in our study included all participants that completed at least one of the 7 timepoints of the bullying victimisation exposure measures from age 4 to 16, and at least one of the 5 timepoints of the depression symptoms outcome measure from age 16.5 to 23. A total of 6782 mother-child dyads were included. Pairwise correlations of the exposure and outcome measures were examined to assess temporal relationships and identify potential multicollinearity that could affect the main analyses (see Tables 1 and 2, and 3 in the supplementary materials).

In longitudinal birth cohort studies, it is typical that missingness relates to observed participant characteristics or study variables as shown in Table 1. Therefore, the missing data here is assumed to be missing at random (MAR). As such, to account for missing data in this analytical sample, we implemented multiple imputation by chained equations, using predictive mean matching via the R package *mice* [27]. Fifty imputed datasets were created, each with twenty iterations, and were analysed directly with SLCMA which pools estimates across imputations according to Rubin’s rules [28].

**Table 1:** Summary statistics of exposures, outcomes, and covariates

	Analytical sample			Non-analytical sample		
	*N* = 6782	Mean/Proportion	Standard Deviation	*N* = 8229	Mean/Proportion	Standard Deviation
Participant sex at birth						
Male	2905	42.8%	-	4785	58.0%	-
Female	3877	57.2%	-	3471	42.0%	-
Maternal educational attainment						
CSE	766	12.1%	-	1759	28.6%	-
Vocational	508	8.0%	-	721	11.7%	-
0 level	2186	34.5%	-	2137	34.8%	-
A level	1722	27.2%	-	1071	17.4%	-
Degree	1149	18.1%	-	459	7.5%	-
Total responders	6331	-	-	6147	-	-
Family financial difficulties						
8 months postpartum	6138	2.885	3.446	5108	3.581	3.756
21 months postpartum	5948	2.764	3.405	4304	3.642	3.829
33 months postpartum	5866	2.753	3.472	3802	3.677	3.889
Maternal mental health						
EPDS score at 32 weeks gestation	6182	6.570	4.848	6003	7.620	5.279
EPDS score at 21 months postpartum	5997	5.447	4.635	4382	6.104	4.986
Suicide attempts at 33 months postpartum	5863	0.003	0.051	3814	0.008	0.090
Participant behavioural difficulties score						
3.6 years	5996	12.210	5.473	4011	13.021	6.077
Bullying victimisation (1 = not true, 2 = somewhat true, 3 = certainly true)						
4 years	5882	1.100	0.329	3569	1.125	0.361
7 years	5452	1.183	0.432	2671	1.219	0.458
8 years	5528	1.229	0.462	2266	1.270	0.487
9 years	5596	1.244	0.485	2200	1.284	0.532
11 years	5371	1.231	0.480	1721	1.264	0.526
13 years	5305	1.219	0.478	1539	1.261	0.540
16 years	4701	1.089	0.329	710	1.092	0.342
SMFQ score						
16.5 years	4883	5.862	5.600	110	7.945	6.793
17.5 years	4268	6.582	5.256	225	6.604	5.123
21 years	3207	5.662	5.542	96	6.854	6.500
22 years	3692	6.196	5.521	227	6.189	5.644
23 years	3744	6.991	6.025	277	7.675	6.407

*Financial difficulty was assessed using a scale such that a score of 15 indicates the most financial difficulty. Maternal mental health was determined by two measures: (1) The Edinburgh Postnatal Depression Scale (EPDS) and (2) suicide attempts. Scores above 13 on the EPDS indicates likely depression. Suicide attempts were measured by a score such that 0 indicates no suicide attempts, 1 indicates an attempt. The participant behavioural difficulties score was derived from Revised Rutter Parent Scale for Preschool Children (RRPSPC) such that a sum score between 0 and 56 is compiled, with a score of 56 indicating high levels of behavioural difficulties*

## Measures

### Questionnaires

Bullying victimisation was assessed using the following item from the mother-reported Strength and Difficulties Questionnaire (SDQ) - “has your child been bullied or picked on in the past six months.” The SDQ item is rated on a 3-point Likert scale either 0 (“not true”), 1 (“somewhat true”), and 2 (“certainly true”). The timepoints of the SDQ item included in this study are at ages 4, 7, 8, 9, 11, 13, and 16. While the SDQ has been extensively validated as a screening tool for emotional and behavioural difficulties in children [29–31], formal psychometric validation of this specific bullying item is limited. Correlations of child-reported Bullying and Friendship Interview Schedule (BFIS) at ages 8.5 and 10.5 by the single SDQ mother-reported item at ages 8, 9, and 11 showed modest parent-child agreement (see Table 4 in supplementary materials).

To assess depression symptoms, we selected the child-reported Short Mood and Feelings Questionnaire (SMFQ), a 13-item questionnaire which measures the affective and cognitive symptoms of depression occurring in the past 2 weeks [32, 33] and has been validated in clinical and non-clinical samples [34–38]. Each item is scored on a 3-point Likert scale between 0 (“Not True”), 1 (“Sometimes”), and 2 (“True”) and the resulting sum score ranges between 0, not depressed, and 26, highly depressed. The timepoints of the SMFQ are at ages 16.5, 17.5, 21, 22, and 23. The SMFQ in this sample has good reliability at all the timepoints with internal consistency assessed by ordinal Cronbach alpha > 0.94.

### Developmental windows

To test for sensitive periods of bullying victimisation, the bullying exposure as assessed by the SDQ was categorised into four developmental windows based on established developmental periods: early childhood (0–5 years), middle childhood (6–10 years), early adolescence (11–13 years) and late adolescence (14–18 years). The bullying measures were combined such that the 4-year-old timepoint represents early childhood, the 7, 8, and 9-year-old timepoints represent middle childhood, the 11 and 13-year-old timepoints represent early adolescence and the 16-year-old timepoint represents late adolescence. Sensitivity analyses were conducted to test whether the different number of timepoints per period resulted in consistent findings (see Fig. 2 in supplementary materials).

### Operationalisation of the timing hypotheses

The accumulation hypothesis was operationalised by summing bullying exposures across all measured timepoints to capture the total burden of exposure. For the recency hypothesis, a weighted sum of bullying exposures was calculated, assigning greater weight to more recent timepoints to reflect the potential stronger influence of proximal experiences. The sensitive period hypothesis treated bullying exposure within a developmental window as a distinct continuous variable, comprised of the sum of bullying victimisation score during that period, allowing the model to identify whether specific developmental windows exhibit heightened sensitivity to bullying victimisation.

### Structured life course modelling

To examine the relationship between the timing of bullying victimisation during childhood and depression symptoms in emerging adulthood, a structured life course modelling approach (SLCMA) was applied. SLCMA tests pre-specified life course timing theories in exposures for later life outcomes [39]. SLCMA uses least angle regression (LARS) to implement the least absolute shrinkage and selection operator (LASSO) to identify the single hypothesis, or combination of hypotheses, that has the most parsimonious explanation for the variation in the observed outcome [28, 40, 41]. When fitting the model, the LASSO systematically adds a variable, in this case, a timing theory variable, and either selects it or shrinks it out of the model depending on its contribution [39, 40]. The number of timing theory variables included in the final model was then determined by examining elbow plots, which plot the explained variance as a function of the number of timing theory variables. Variable selection is based on where an elbow is present in the plot, which indicates diminishing returns in variance explained with added variables. As such, the variables before the elbow and at the elbow are selected as contributing the most to the model. Once the variables were selected, selective inference, a post-selective inference method, is employed to obtain coefficients, confidence intervals, and p-values. Selective inference is recommended to minimise family-wise error rate [42]. With SLCMA, the accumulation, recency, and the sensitive period hypotheses can thus be compared to determine which hypothesis (or set of hypotheses) best explains the outcome in this dataset.

### Covariates

To adjust for confounding, covariates were included in the SLCMA models based on prior research identifying key risk factors associated with both bullying and depression. Maternal psychopathology was included given its well-established influence on child mental health and increased risk of bullying victimisation [43, 44] and was determined by two measures: (1) The Edinburgh Postnatal Depression Scale (EPDS) and (2) suicide attempts. Scores above 13 on the EPDS indicate likely depression. Suicide attempts were measured by a score such that 0 indicates no suicide attempts and 1 indicates an attempt. Family socioeconomic status was controlled for due to its association with increased bullying risk and adverse mental health outcomes [45, 46] and was determined by a financial difficulty scale with scores of 15 indicating the most financial difficulty. The study child’s behavioural problems prior to bullying exposure were included as a potential marker for pre-existing psychopathology which is linked to vulnerability to being bullied. The participant behavioural difficulties score was derived from the Revised Rutter Parent Scale for Preschool Children (RRPSPC) such that a sum score between 0 and 56 is compiled, with a score of 56 indicating high levels of behavioural difficulties. Finally, the study child’s gender was included due to known sex differences in bullying depression prevalence during adolescence [47].

All analyses were carried out in R version 4.2.2.

## Results

### Sample characteristics

The analytical sample (n = 6,782) is predominantly White (96%) and includes 43% males and 57% females. 51.6% of the sample, reported being “somewhat bullied” or “certainly bullied” at some point across ages 4 to 16 years (for summary statistics, see Table 1). Participants lost to follow-up were more likely to be male and have mothers with lower educational attainment. They also tended to come from families reporting greater financial difficulties and higher maternal mental health symptoms.

### Model selection

**Fig. 1:**
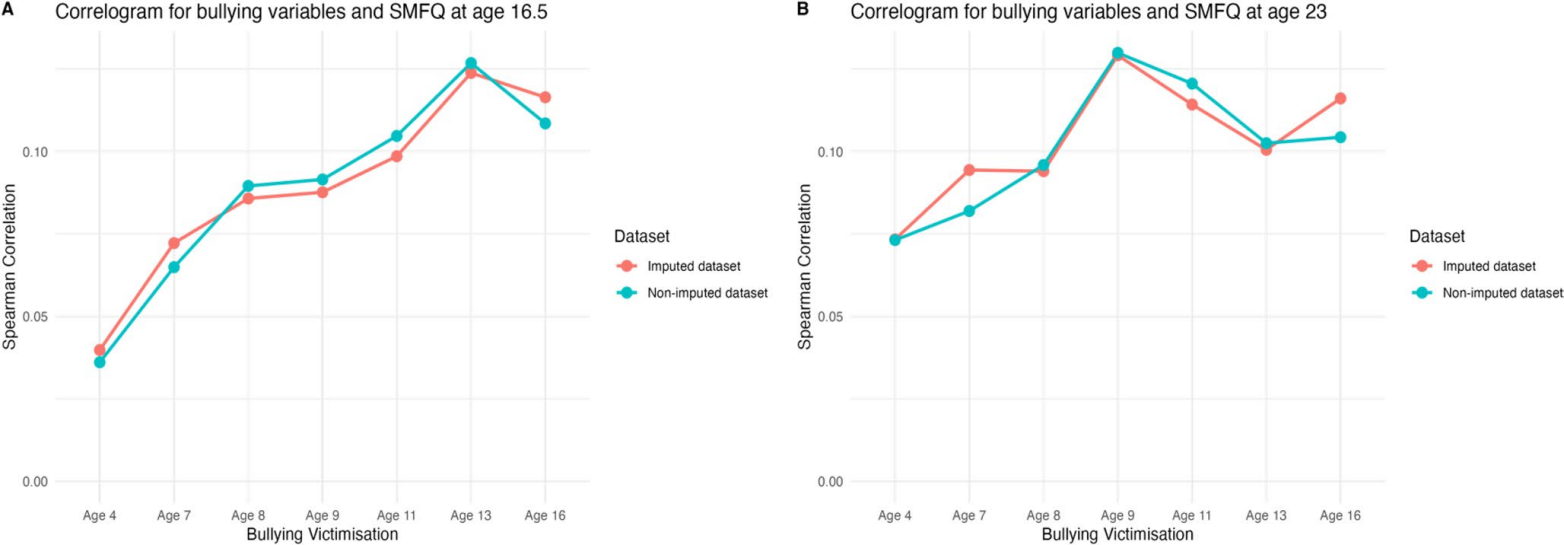
Correlograms for Spearman correlations between bullying victimisation and SMFQ scores at age 16.5 (panel **A**) and age 23 (panel **B**)

To describe the relationship between bullying victimisation and depression symptoms, two correlograms were generated to illustrate how the associations change over time. Figure 1**panel A** presents the Spearman correlations between bullying victimisation between ages 4 and 16, and the SMFQ scores at age 16.5, while **panel B** depicts the same correlations for SMFQ scores at age 23. Notably, both figures show strong similarities between the imputed and non-imputed datasets, indicating that missingness is unlikely to substantially impact findings. Additionally, the correlations tend to increase as the bullying experience occurs closer to the SMFQ assessment age, illustrating the recency effect. This trend remains consistent across both SMFQ timepoints, indicating that while early bullying has a lasting impact, more recent experiences may be particularly influential. When comparing both correlograms, the slope in panel A appears less steep than in panel B, suggesting that the recency trend lessens the further the outcome is from the exposure. This is consistent with the results from post-hoc analyses conducted to investigate how far the recency trend reaches when increasing the amount of time between the last exposure timepoint and the outcome (see Fig. 1 in supplementary materials).

**Fig. 2:**
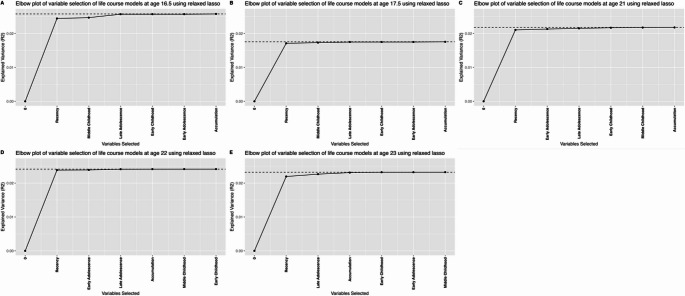
Grid of elbow plot for least angle regression variable selection testing life course models of exposure to bullying victimisation during childhood and depression symptoms at age 16.5, 17.5, 21, 22, and 23

When tested formally, the recency model was chosen at all outcome timepoints (ages 16.5, 17.5, 21, 22, and 23) as the hypothesis most supported by data (see the elbow plots in Fig. 2). After selecting for the recency variable per the elbow plots, post-selective inference was conducted to further confirm the model choice. It revealed that the recency model is supported for all outcome times with *p* < 0.001 (see Table 2). The explained variance accounted for in the models ranged from 1.6% to 2.3%. The coefficients of the recency model were such that exposure to bullying was associated with an increase in SMFQ score for all timepoints of bullying and SMFQ, with a greater increase for exposure to bullying at older ages (i.e. more proximal to the outcome measurement). For example, as can be seen in Table 2, exposure to bullying at age 4 was associated with an increase of 0.188 (95%CI: 0.160–0.216) in SMFQ score at age 16.5, while at age 16 the increase was 0.753 (95% CI: 0.640–0.867).

**Table 2:** Coefficients of the selective inference for recency model selection at illustrative exposure ages 4, 8, 11, and 16

		Exposure at 4		Exposure at 8		Exposure at 11		Exposure at 16	
Outcome age	*p*-value	Coefficient	Confidence Intervals	Coefficient	Confidence Intervals	Coefficient	Confidence Intervals	Coefficient	Confidence Intervals
16.5	< 0.001	0.188	0.160–0.216	0.376	0.320–0.433	0.518	0.440–0.596	0.753	0.640–0.867
17.5	< 0.001	0.149	0.122–0.176	0.298	0.244–0.352	0.410	0.335–0.485	0.596	0.488–0.705
21	< 0.001	0.174	0.146–0.203	0.349	0.292–0.400	0.480	0.402–0.559	0.699	0.585–0.814
22	< 0.001	0.184	0.156–0.212	0.369	0.312–0.425	0.508	0.430–0.585	0.739	0.625–0.851
23	< 0.001	0.193	0.162–0.224	0.386	0.324–0.448	0.531	0.446–0.616	0.772	0.649–0.851

### Post-hoc and sensitivity analyses

A post-hoc set of analyses was conducted to investigate if bullying at age 16 was driving the recency effect in the main analyses. SLCMA models were conducted for bullying at ages 4, 7, 8, 9, and 11 and depression symptoms at ages 12.5, 13.5, 16.5, 17, 21,22, and 23. The recency model was chosen for all outcome timepoints except for at age 23, at which point, accumulation was selected (see Fig. 1 in supplementary materials).

Sensitivity analyses were also conducted with different developmental windows for the exposure to test whether the categorisation of windows may have influenced the main results. The recency hypothesis was selected for all models (see Fig. 2 in supplementary material).

Finally, sensitivity analyses were conducted to compare categorising the bullying measure as continuous versus binary. The recency hypothesis was selected for all models (see **Fig. **3 in supplementary material).

## Discussion

We found that depression symptoms in late adolescence and emerging adulthood were largely explained by the recency of exposure to bullying victimisation, rather than accumulation of bullying or sensitive periods during development. This suggests that more recently occurring exposures, relative to the time at which depression symptoms were measured, are more strongly associated with the outcome than distal exposures.

These results are partially consistent with the Singham [22] paper which found a decrease of depression symptoms after 2 years with a complete fade out after 5 years following the exposure to bullying. This is also consistent with the results of a meta-analysis on quasi-experimental evidence of short- and long-term consequences of bullying victimisation which demonstrated that the effect of bullying victimisation on internalising disorders sharply decreased as time elapsed from the exposure [12]. Taken together, those findings suggest that, although bullying does incur clear, immediate risks to mental health, these risks can be mitigated with time, pointing to the potential for resilience in children experiencing bullying victimisation.

In addition to anti-bullying interventions aiming to prevent the occurrence of bullying in the first place, these findings suggest that interventions fostering resilience post bullying are warranted. Such interventions should aim to provide scaffolding for the recovery process as early as possible once bullying is identified, and possibly for as long as required to return to baseline mental health.

We note that, while the recency hypothesis was supported at all time points, some questions remain regarding the patterns of correlation between bullying exposure and depression outcomes. As illustrated in Table 2, the size of effects observed across ages is similar for ages 16.5 and 23, while we would expect decreased effect sizes at age 23 given greater time elapsed from the exposure to bullying. This could be explained by better reliability of the outcome measure in emerging adulthood as better reliability can lead to larger effect sizes, thereby possibly compensating the aforementioned expected decrease in effect sizes. However, the reliability of the SMFQ was high at all time points. It is also possible that residual confounding by time-invariant confounders (e.g. genetic confounding [48]) may contribute to persisting associations in the long term. Finally, it is possible that the reliability of the bullying victimisation variable gradually increases from early childhood to adolescence, generating a pattern of correlation consistent with the recency hypothesis. Future research should explore these alternative explanations with different measures and in different cohorts.

### Strengths and limitations

This study has several strengths. We employed a novel statistical approach that allowed us to systematically investigate the pre-specified timing models of accumulation of risk, recency, and sensitive periods. SLCMA has allowed us to go beyond investigating dualities such as exposed versus not-exposed or a single timing hypothesis [28]. Furthermore, we used data from a longitudinal birth cohort which reduces recollection bias given that measures are collected prospectively across the lifetime. Additionally, using a mother-reported measure for the exposure and a participant-reported questionnaire for the outcome helps mitigate shared-rater bias [12].

However, the study has a few limitations. The ALSPAC data is located in the former county of Avon in the United Kingdom and is predominantly White, middle class, and born in the 90s, limiting generalisation. Furthermore, we selected mother-reported bullying victimisation via the SDQ item as it is available repeatedly across childhood and adolescence, which was essential to the aim of this study. However, using a single-item measure for bullying may increase measurement error and underestimate associations with mental health.

As the item is mother-reported, it is also subject to reporting bias especially for later timepoints; a mother may be best placed to report on their child’s bullying experiences at age 4, but this may not be the case by age 16 as their child becomes increasingly independent. However, such an increasing bias would result in increased measurement error and a likely corresponding decrease in the association between bullying in adolescence and later depression, which would run against the selection of the recency hypothesis.

Additionally, while the SDQ item is widely used in population studies and facilitates repeated assessment across development, it has not been specifically validated for detailed measurement of bullying victimisation. Its brevity and general wording mean it cannot capture the heterogeneity of bullying experiences (e.g., overt vs. relational victimisation), which may differentially influence depression risk. However, we chose to retain this item as we needed a measure repeated across wide developmental windows to formally test developmental hypotheses. Future studies employing a well-validated, multi-item measure of bullying, repeated across multiple timepoints across childhood and adolescence, would allow for more precise assessment of exposure and subtype-specific effects.

More frequent measures of bullying at stable intervals (i.e., every few months) throughout childhood and adolescence would be ideal to thoroughly investigate the question of timing of bullying on later depression, including other relationships such as chronicity of the exposure. However, this data is typically unavailable within longitudinal studies over long developmental periods.

Furthermore, the developmental windows specified in the study followed classical cut off ages to delineate school transitions. These windows are not empirically supported as established sensitive periods in developmental psychology and should be interpreted cautiously, especially given the results point towards recency and not sensitive periods.

Although we controlled for some covariates, SLCMA is not a causal inference method and focuses mainly on testing developmental hypotheses. As such, estimates should not be interpreted causally as they might be confounded by other types of covariates (i.e., other adversities, or genetic confounding [48]). Future research should investigate the combined and independent effects of multiple adversities alongside bullying victimisation.

The effect sizes appear to be modest with values between 1.6% and 2.3%. Such values are to be expected in developmental psychology research, and more so in longitudinal studies with large time spans [49]. These values are well within the expected range for SLCMA studies with similar exposures and outcomes [50].

Finally, although SLCMA rigorously tests for timing patterns, it comes with some limitations, notably that it cannot control for unobserved confounding and time-varying covariates, precluding a causal interpretation of our findings.

## Conclusion

The findings suggest that the association between bullying victimisation and depression symptoms is largely driven by recency. This aligns with previous research indicating that the negative psychological associations with bullying may diminish over time, reflecting a pattern of resilience. Overall, the results underscore the importance of addressing the acute impact of bullying, emphasising that early intervention should be prioritised to foster resilience and thus mitigate its consequences on subsequent depression.

While the potential for long-term resilience is positive for children’s outlook, this does not diminish the immediate risks that bullying poses to mental health. In addition, future research should aim to disentangle whether sustained associations between bullying and adult outcomes result from bullying itself or from other unmeasured factors.

Ultimately, while time may help lessen the psychological burden of past bullying experiences, the priority should remain on preventing victimisation in the first place as well as providing timely support for those affected.

## Supplementary information

Below is the link to the electronic supplementary material.

Supplementary File 1 (DOC 484 KB)

## Data Availability

Access to ALSPAC data is not public and it is provided by the University of Bristol. Information on how to apply for access to ALSPAC data is provided here: (https://www.bristol.ac.uk/alspac/researchers/access).
