# Supplementary material for: Timing effects in the association between childhood and adolescent bullying victimisation with late adolescence and emerging adulthood depressive symptoms
Source: Eur Child Adolesc Psychiatry. 2026 Mar 23;35(7):2179–88. doi: 10.1007/s00787-026-03011-9 (PMC13427968; doi:10.1007/s00787-026-03011-9)
Supplement: Supplementary file 1 — (DOC 484 KB) [file 787_2026_3011_MOESM1_ESM.docx]

**Supplementary materials**

**Post-hoc analyses**


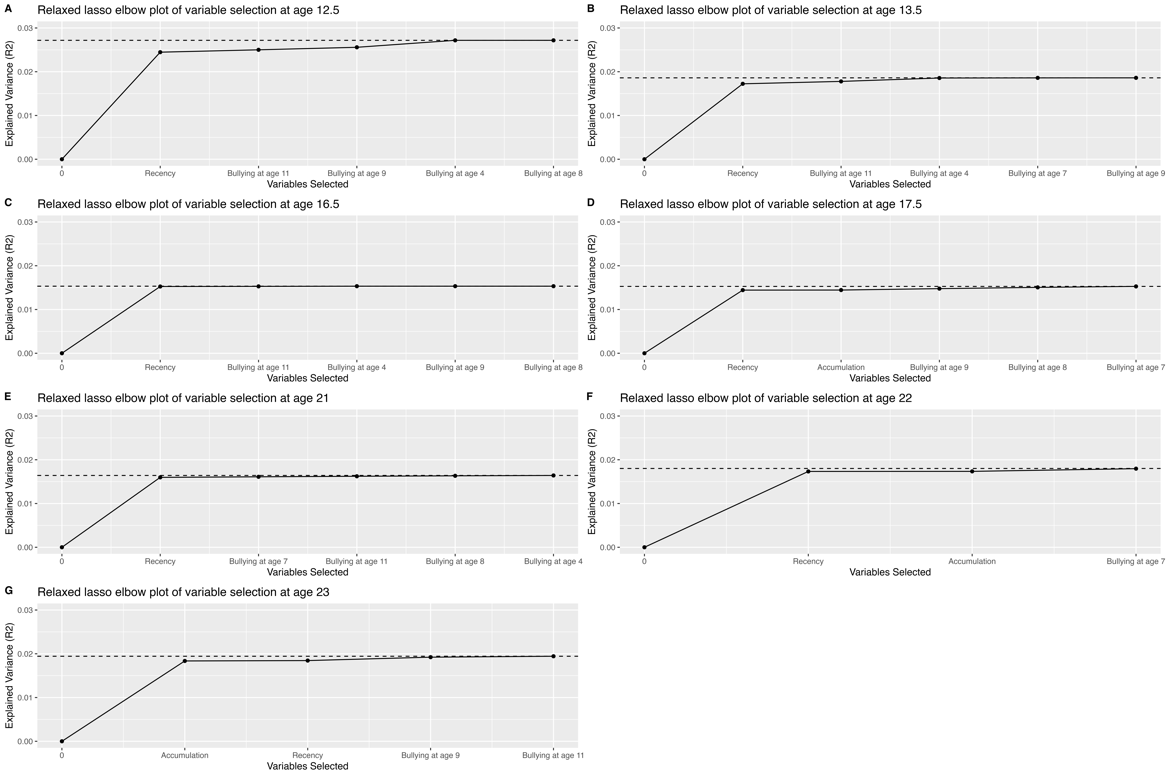
To assess the effect of the 16-year assessment, and verify whether the recency hypothesis still held for bullying victimisation in childhood, we repeated SLCMA analyses for bullying victimisation at ages 4, 7, 8, 9, and 11 and depression symptoms from ages 12.5, 13.5, 16.5, 17.5, 21, 22, and 23. The recency model was chosen at most outcome timepoints (ages 12.5, 13.5, 16.5, 17.5, 21, and 22) as the hypothesis most supported by the data except for the outcome at age 23, at which accumulation was chosen. After selecting for the recency variable as per the elbow plots, post-selective inference was conducted to further confirm the model choice. It revealed that the recency model is statistically significant for all outcome times with p < 0.001. The explained variance accounted for in the models spanned between 1.4% and 2.4%. These results further confirm that the prolonged selection for recency that was observed in the main results is not an isolated case as these results also indicate a similar pattern, until accumulation was selected at outcome age of 23.

**Figure 1** Grid of elbow plots of model selection for bullying between ages 4 and 11 and SMFQ scores at ages 12.5, 13.5, 16.5, 17.5, 21, 22, and 23

**Sensitivity analyses**

**Testing different categorisation of sensitive periods**

Using the imputed sample, sensitivity analyses were conducted, varying sensitive period ranges to test whether the categorisation of sensitive periods may have swayed the main analyses. To maintain equal timepoints in each sensitive period range, two developmental periods were categorised (1) middle childhood, including timepoints 7, 8, and 9, and (2) adolescence, including timepoints 11,13, and 16. The recency model was chosen at all outcome timepoints (ages 16.5, 17.5, 21, 22, and 23) as the hypothesis most supported by data (see the elbow plots in **Figure 2**). After selecting for the recency variable per the elbow plots, post-selective inference was conducted to further confirm the model choice. It revealed that the recency model is statistically significant for all outcome times with p < 0.001. The explained variance accounted for in the models spanned between 1.5% and 2.2%. The results largely match the main analyses, regardless of the categorisation of sensitive period ranges.

**
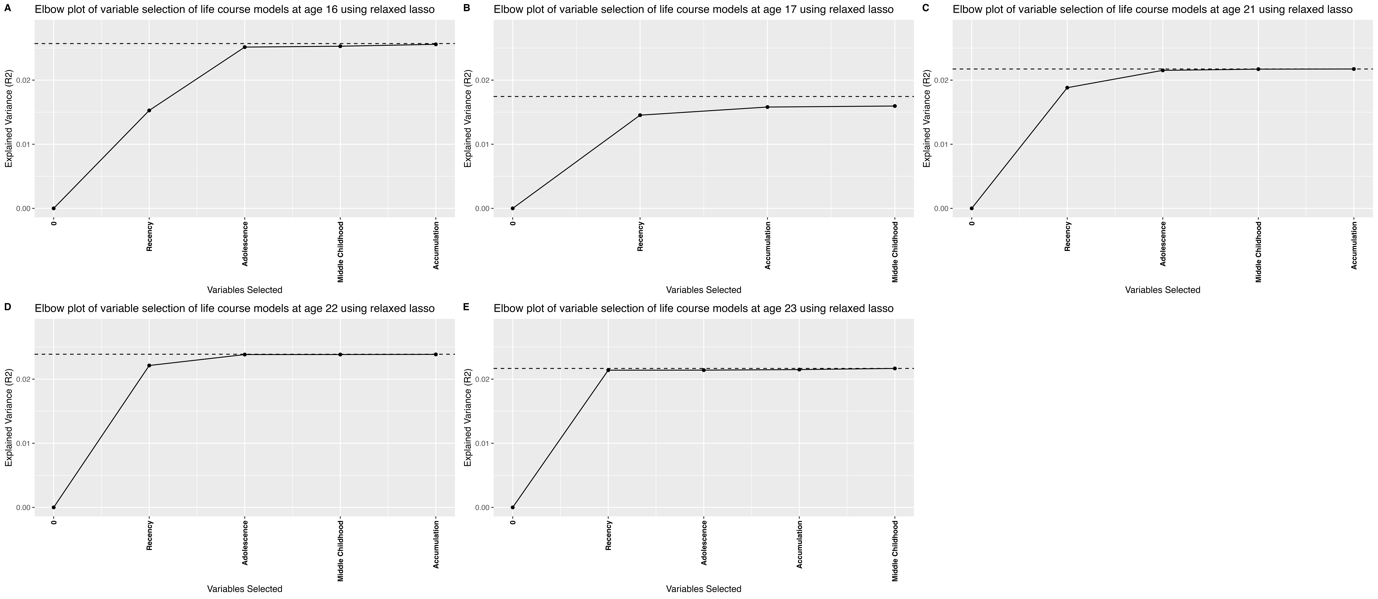
**

**Figure 2** Grid of elbow plots of model selection for bullying between ages 4 and 16 and SMFQ scores at ages 16.5, 17.5, 21, 22, and 23

**Testing bullying as a binary measure**

**
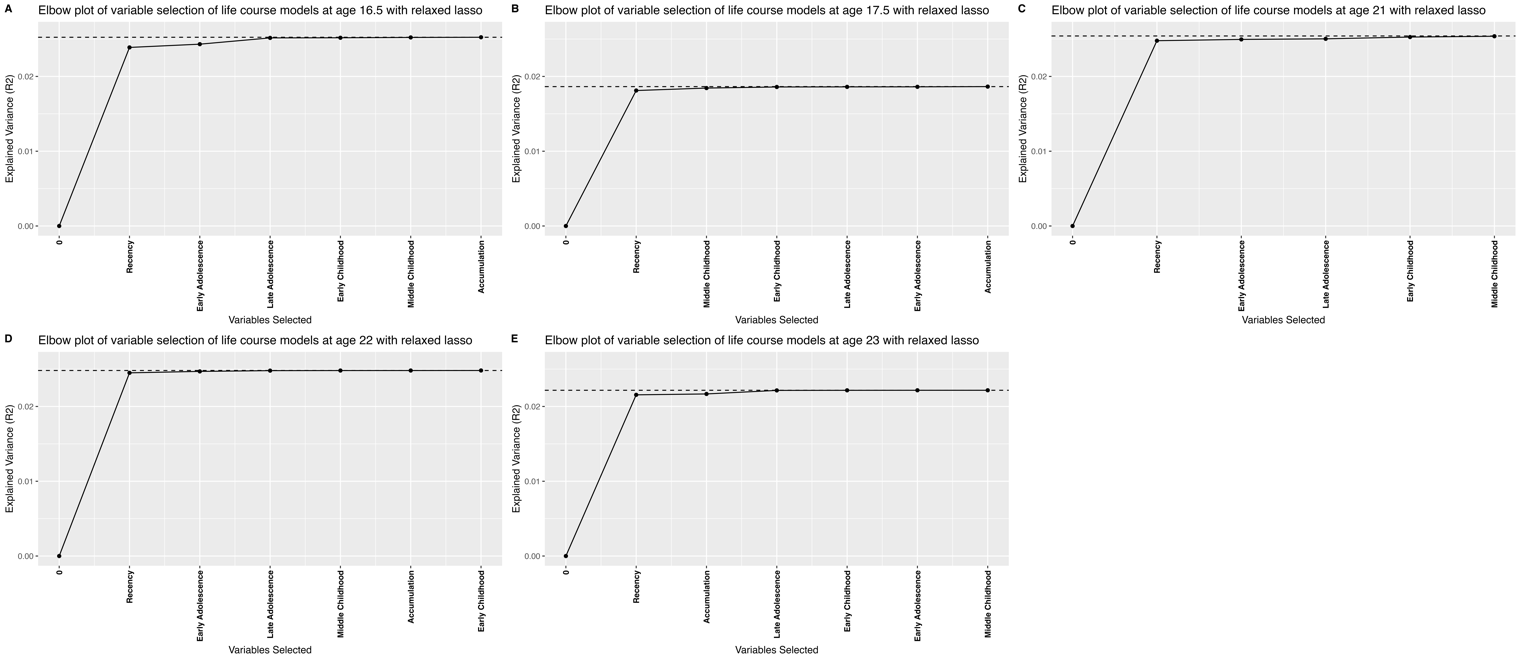
**Using the imputed sample, sensitivity analyses were conducted, with binarising the bullying measure such that responses of “never true” represented no bullying occurrence and responses of “somewhat true” and “always true” were categorised as evidence of bullying occurrence. The same sensitive periods as the main analyses were maintained. The recency model was chosen at all outcome timepoints (ages 16.5, 17.5, 21, 22, and 23) as the hypothesis most supported by data (see the elbow plots in **Figure 3**). After selecting for the recency variable per the elbow plots, post-selective inference was conducted to further confirm the model choice. It revealed that the recency model is statistically significant for all outcome times with p < 0.001. The explained variance accounted for in the recency models spanned between 1.0% and 2.3%. The results largely match the main analyses, regardless of the categorisation of sensitive period ranges.

**Figure 3** Grid of elbow plots of model selection for bullying between ages 4 and 16 and SMFQ scores at ages 16.5, 17.5, 21, 22, and 23

**Complete case correlations for study data**

Table 1 Correlations of bullying exposure by bullying exposure as assessed by the Strengths and Difficulties Questionnaire (SDQ) using complete observations

|  | **SDQ 4 yrs** | **SDQ 7 yrs** | **SDQ 8 yrs** | **SDQ 9 yrs** | **SDQ 11 yrs** | **SDQ 13 yrs** | **SDQ 16 yrs** |
| --- | --- | --- | --- | --- | --- | --- | --- |
| **SDQ 4 yrs** | 1 | 7.5e-40 | 1.7e-25 | 4.6e-23 | 4.4e-23 | 3.9e-10 | 1.6e-11 |
| **SDQ 7 yrs** | .18 | 1 | 1.1e-132 | 1.3e-126 | 3.8e-79 | 1.9e-54 | 3.4e-28 |
| **SDQ 8 yrs** | .15 | .37 | 1 | 2.4e-169 | 6.1e-94 | 2.1e-70 | 9.9e-36 |
| **SDQ 9 yrs** | .16 | .35 | .38 | 1 | 1.3e-192 | 4.8e-127 | 3.4e-34 |
| **SDQ 11 yrs** | .14 | .29 | .29 | .41 | 1 | 7.1e-226 | 8.1e-62 |
| **SDQ 13 yrs** | .08 | .25 | .26 | .34 | .41 | 1 | 7.7e-85 |
| **SDQ 16 yrs** | .10 | .17 | .18 | .19 | .26 | .28 | 1 |

Note. Lower triangle = Pearson correlation coefficients (r).
Upper triangle = two-tailed p-values in scientific notation.

Pearson correlations were computed using complete cases datasets.

N ranges from 4161 and 5882.

We note that all values are significant at p < .001.

Table 2 Correlations of SMFQ score by SMFQ score using complete observations

|  | **SMFQ 16.5 yrs** | **SMFQ 17.5 yrs** | **SMFQ 21 yrs** | **SMFQ 22 yrs** | **SMFQ 23 yrs** |
| --- | --- | --- | --- | --- | --- |
| **SMFQ 16.5 yrs** | 1 | 3.9e-204 | 7.1e-98 | 2.8e-96 | 7.8e-97 |
| **SMFQ 17.5 yrs** | .54 | 1 | 1.1e-103 | 6.3e-123 | 1.5e-111 |
| **SMFQ 21 yrs** | .39 | .42 | 1 | 6.8e-267 | 1.2e-205 |
| **SMFQ 22 yrs** | .43 | .43 | .62 | 1 | < 2.0e-308 |
| **SMFQ 23 yrs** | .40 | .39 | .58 | .67 | 1 |

Note. Lower triangle = Pearson correlation coefficients (r).
Upper triangle = two-tailed p-values in scientific notation.

Pearson correlations were computed using complete cases datasets.

N ranges from 2260 to 4268.

We note that all values are significant at p < .001.

Table 3 Correlations of SMFQ score by bullying victimisation exposure (SDQ) using complete observations

|  | **SMFQ 16.5 yrs** | **SMFQ 17.5 yrs** | **SMFQ 21 yrs** | **SMFQ 22 yrs** | **SMFQ 23 yrs** |
| --- | --- | --- | --- | --- | --- |
| **SDQ 4 yrs** | .09 | .07 | .05 | .06 | .07 |
| **SDQ 7 yrs** | .06 | .06 | .10 | .11 | .05 |
| **SDQ 8 yrs** | .12 | .14 | .14 | .13 | .11 |
| **SDQ 9 yrs** | .15 | .15 | .14 | .14 | .13 |
| **SDQ 11 yrs** | .16 | .12 | .15 | .15 | .12 |
| **SDQ 13 yrs** | .17 | .16 | .19 | .15 | .12 |
| **SDQ 16 yrs** | .09 | .11 | .10 | .12 | .11 |

Note. We note that all values are significant at p < .001

Pearson correlations were computed using complete cases datasets.

N ranges from 2468 to 4233.

**Correlations using imputed datasets**

Bullying and Friendship Interview Schedule (BFIS) was administered to the participant at ages 8.5 and 10.5. We compiled a correlations table of the BFIS scores at ages 8.5 and 10.5 with the single SDQ bullying item at ages 8, 9, and 11. The results were as expected in multi-informant assessments, where parent-child agreement is typically modest. The correlations were positive and in the small-to-moderate range, indicating that the SDQ bullying item captures overlapping information with the child-reported measures while reflecting the known limitations of single-item, parent-reported assessments. The full correlation matrix is provided below:

Table 4 Correlations of BFIS scores by bullying exposure as assessed by a single SDQ item

|  | **BFIS 8.5 yrs** | **BFIS 10.5 yrs** | **SDQ 8 yrs** | **SDQ 9 yrs** | **SDQ 11 yrs** |
| --- | --- | --- | --- | --- | --- |
| **BFIS 8.5 yrs** | 1 | 8.2e-77 | 5.2e-35 | 2.7e-36 | 1.2e-26 |
| **BFIS 10.5 yrs** | .346 | 1 | 2.645e-19 | 4.375e-45 | 1.1e-44 |
| **SDQ 8 yrs** | .188 | .142 | 1 | 1.4e-107 | 1.7e-54 |
| **SDQ 9 yrs** | .212 | .219 | .383 | 1 | 1.5e-80 |
| **SDQ 11 yrs** | .175 | .225 | .300 | .414 | 1 |

Note. Lower triangle = Pearson correlation coefficients (r).
Upper triangle = two-tailed p-values in scientific notation.

Pearson correlations were computed using imputed datasets.

We note that all values are significant at p < .001.

**Covariates for SLCMA models and auxiliary variables for multiple imputations**

|  | Variable ID | Variable | Timepoint |
| --- | --- | --- | --- |
|  | kz021 | Participant sex at birth |  |
| Social class | mz028b | Maternal age at birth of study child |  |
|  | c645a | Maternal highest education level | 32 weeks’ gestation |
|  | f805, g840, h735, | Family financial difficulties | 8 months post-partum, 21 months post-partum, 33 months post-partum |
|  | c_mcamsis_m | Paternal CAMSIS score |  |
|  | c_fcamsis_m | Maternal CAMSIS score |  |
| Maternal psychopathology pre-birth | c574 | Crown-Crisp Experiential Index (anxiety) | 32 weeks’ gestation |
|  | c601 | Maternal Edinburgh Postnatal Depression Scale | 32 weeks’ gestation |
|  | b597 | Maternal suicide attempts | 32 weeks’ gestation |
| Maternal psychopathology post-partum | g291 | Maternal Edinburgh Postnatal Depression Scale | 21 months post-partum |
|  | g269 | Crown-Crisp Experiential Index (anxiety) | 21 months post-partum |
|  | h238 | Maternal suicide attempts | 33 months post-partum |
| Child’s pre-existing behavioural and emotional difficulties | kj647 | Revised Rutter Parental Scale for Preschool Children – Total Difficulties Score | 42 months |
